# Retraction Note: The role of acute care surgeons in treating rib fractures—a retrospective cohort study from a single level I trauma center

**DOI:** 10.1186/s12893-023-01915-w

**Published:** 2023-01-24

**Authors:** Chia-Cheng Wang, Szu-An Chen, Chi-Tung Cheng, Yu-San Tee, Sheng-Yu Chan, Chih-Yuan Fu, Chien-An Liao, Chi-Hsun Hsieh, Ling-Wei Kuo

**Affiliations:** Department of Trauma and Emergency Surgery, Chang Gung Memorial Hospital, Linkou Medical Center, No. 5, Fuxing St., Guishan District, Taoyuan, 333 Taiwan

**Retraction Note: BMC Surgery (2022) 22:271** **https://doi.org/10.1186/s12893-022-01720-x**

The Executive Editor has retracted this article since the authors were unable to provide the ethics approval documentation for this study. All authors agree to this retraction.

